# Pharmaceutical humanistic care in practice: A pharmacist’s personal journey from technical focus to compassionate care

**DOI:** 10.1017/S1478951526102296

**Published:** 2026-06-05

**Authors:** Feng Xu

**Affiliations:** Department of Pharmacy, Shanghai DeltaHealth Hospital, Shanghai, China

## Introduction

Hospital pharmacy has always been shaped by health policies, technological innovations, and patient needs. In recent years, China’s national volume-based drug procurement policy has dramatically reduced drug prices – folic acid tablets now cost less than $0.004 USD per tablet – leading some to jest that hospital pharmacies have become “increasingly worthless places.” Simultaneously, beginning July 2025, all medication dispensing in China will require comprehensive barcode scanning, substantially increasing pharmacists’ workload.

More daunting is technological disruption: artificial intelligence agents have demonstrated overwhelming advantages in pharmacy practice. In prospective prescription review, AI agents rapidly identify problematic prescriptions; in medication counseling, they provide 24/7 standardized guidance; in evidence synthesis, they aggregate global research in real time. This revolution has fostered pessimistic beliefs that AI understands drugs better than pharmacists and that pharmacists will eventually be replaced.

Examining pharmacists’ actual work conditions reveals additional pressures. Highly educated clinical pharmacists must balance clinical care, teaching, and research responsibilities while navigating key performance indicator assessments. Dispensing pharmacists face tedious window work and patient complaints about waiting times. Meanwhile, regions like Shanghai in China have yet to establish comprehensive pharmacy service fee structures; even where pilot programs exist, fees rarely cover costs, creating a situation where pharmacy services cannot sustain themselves. These factors compound young pharmacists’ loss of professional identity and burnout, with some choosing to “lie flat” or leave the profession entirely.

## A transformative experience: My father’s illness

My awareness of pharmaceutical humanistic care began with a clinical experience involving my father – an experience that fundamentally reshaped my understanding of clinical pharmacy’s core values.

In the early summer of 1993, I worked in the pharmacy department of the First Affiliated Hospital of the First Military Medical University of the People’s Liberation Army of China (now Southern Medical University Affiliated Nanfang Hospital). My primary responsibility was therapeutic drug monitoring, while also participating in research studying novel formulations of the anticancer drug methotrexate. At that time, clinical pharmacy work centered largely on analytical methods and pharmacokinetics; truly engaging with patients at the bedside to understand their medication experiences was exceedingly rare. My perspective then was narrowly focused on maximizing drug efficacy through precise monitoring. Patients’ psychological needs received little attention. This technology-first orientation was profoundly challenged during my father’s treatment.

My father, a retired military officer, was diagnosed with poorly differentiated gastric adenocarcinoma in 1990 and underwent subtotal gastrectomy. In 1993, with disease progression, he traveled to Guangzhou for total gastrectomy – a 6-hour surgery complicated by abdominal adhesions. Upon awakening, though clearly in pain, he adamantly refused analgesics. When his wound dehisced on postoperative day 10, he received pethidine after emergency treatment but thereafter refused nearly all analgesic medications.

Initially, I attributed my father’s medication refusal to 2 factors: first, my earlier dogmatic explanations about analgesic addiction, which left him fearing he might become an addict; second, his traditional belief that real men do not fear pain. It was not until he refused medications to manage chemotherapy side effects (ondansetron, erythropoietin, and granulocyte colony-stimulating factor) that I recognized my misunderstanding. As a retired military officer, my father’s medical expenses were fully covered, yet he explicitly asked physicians to minimize expensive imported drugs, explaining, “All drugs have side effects – better to avoid them when possible, and it saves the country money.”

Indeed, as a pharmacy professional operating from an “all drugs are poisons” perspective, I had thoroughly informed my father about potential side effects. Ultimately, unable to tolerate chemotherapy’s side effects – gastrointestinal failure, persistent diarrhea, and dramatic weight loss – my father grew irritable and died 1-year post-surgery.

Years later, reflecting on this experience, I suddenly understood: my father’s medication refusal was fundamentally a fear of side effects. My objective counseling had remained at the level of risk disclosure, neglecting his psychological vulnerability – I neither helped him understand how to contextualize medication risks nor provided essential emotional support. This realization catalyzed my focus on cancer patients’ psychological states.

## Research findings

This reflection prompted my team to study cancer patients’ psychological states and their correlation with treatment outcomes. Our findings revealed that the comorbidity of depression and anxiety among cancer patients is extremely high, and many patients with significant emotional distress received no psychological intervention. Further investigation identified that highly suggestible individuals – including preschool children, frail women, those with chronic physical complaints, and some highly educated individuals – demonstrated significantly heightened sensitivity to medication side effects. When pharmacy professionals mechanically list all possible adverse reactions, they risk triggering negative expectations, potentially inducing the “nocebo effect” – leading to medication refusal.

Our research also elucidated mechanisms by which psychological states influence pharmacotherapy: depression increases subjectively experienced adverse effects; chronic stress alters neurotransmitter expression, increasing nausea and vomiting risk; and depression-induced stress affects drug-metabolizing enzyme activity, altering pharmacokinetics.

Although our series of studies received multiple science and technology awards, I increasingly recognized that the core of clinical pharmacy lies not in publications or awards, but in transforming professional knowledge into tangible patients benefiting not only to drug efficacy but also to patients’ psychological struggles. From this recognition, we began advocating compassionate pharmaceutical services, promoting clinical pharmacists’ evolution from technical implementers to humanistic caregivers.

## The framework of humanistic pharmacy

To achieve this transformation, we must first clarify the concepts of humanistic pharmacy and pharmaceutical humanistic care. The Chinese character for humanism first appeared in the *I Ching* (Book of Changes), emphasizing human value and dignity.

Humanistic pharmacy takes a human-centered approach, examining pharmaceutical issues through a humanistic lens. It encompasses pharmacy history, pharmacists’ spiritual legacy, and the impact of patient psychology on medication use – spanning pharmaceutical education, research, management, and clinical practice.

It is crucial to understand that pharmaceutical humanistic care is not merely polite service. Rather, it represents clinical pharmacists’ capacity to stand in patients’ shoes, using empathic communication to help resolve medication problems and alleviate psychological distress, ultimately improving treatment adherence and healthcare experience.

Practicing pharmaceutical humanistic care requires 3 essential qualities. First, compassion and respect: caring for patients regardless of status, respecting patients’ values and choices, and learning to tolerate different opinions and accept challenging situations. Second, honesty and humility: neither exaggerating efficacy nor concealing risks, using down-to-earth expressions to help patients understand treatment rationale. Third, gratitude: recognizing that patients realize pharmacists’ professional value.

Five principles guide practice: the acceptance principle (accept patients’ emotions without judgment); the equality principle (maintain patients’ dignity); the accommodation principle (consider ethnic and religious backgrounds); the risk disclosure principle (use positively suggestive language); and the economic consideration principle (address medications’ financial toxicity).

## Cultivating humanistic competencies

In an era where AI dominates technical services, humanistic care competency has become clinical pharmacists’ core competitive advantage – irreplaceable by artificial intelligence and key to addressing burnout.

Humanistic care competency encompasses 4 dimensions. Communication competency: conveying respect through professional skills. Understanding competency: uncovering hidden needs from patients’ symptoms and context, recognizing that medication refusal may stem from financial pressure rather than side effect fears. Influence competency: using humanistic principles to impact patients, teams, and systems. Empathy competency: deeply comprehending patients’ emotional experiences and responding appropriately.

In operating our pharmacy clinic, we discovered that patients rarely ask about drug half-life. Their core needs are understanding and reassurance. Even when encountering questions, we cannot immediately answer, honestly telling patients “I’ll review the literature and contact you tomorrow” builds more trust than vague responses. While AI can rapidly retrieve literature and calculate doses, it cannot comprehend patients’ confusion, anxiety, or medication difficulties – all requiring pharmacists’ humanistic attention.

Based on our practice, we propose 5 pathways for cultivating humanistic competency.

First, systematically build humanistic knowledge foundations. Clinical pharmacists should engage with philosophy, literature, history, and ethics. The goal is to elevate emotional awareness – developing critical thinking to avoid black-and-white recommendations, deepening understanding of human nature to avoid treating patients merely as disease vehicles.

Second, focus on specialized humanistic pharmacy literature. Pharmacists should study pharmacy history, pharmacist biographies, ethics, and communication skills, inheriting the spirit of respecting science to help humanity.

Third, learn from senior pharmacists’ humanistic spirit. Senior pharmacists’ practical experience provides living textbooks. In Shanghai, we have organized Pharmacy and Humanities student activities, inviting nationally renowned experts to share their pharmaceutical journeys. Their experiences – accompanying patients on rounds, covering medication costs for impoverished patients – profoundly influence younger generations.

Fourth, deepen social practice and clinical immersion. Participating in multidisciplinary teams helps pharmacists witness patients’ treatment suffering firsthand, understanding that medication side effects are not merely cold text on paper, but patients’ lived experiences. Community volunteer services teach down-to-earth communication approaches.

Finally, cultivate humanistic awareness through writing. Practice reflection journals document success stories and regretful moments. Humanistic writing must derive from authentic experience, not AI generation – AI cannot capture details like a father’s eyes when refusing analgesics.

## Implications

Nearly 40 years of practice have yielded 4 essential insights.

First, warmth is pharmacy’s core essence. Becoming a compassionate pharmacist requires not high intelligence but high emotional intelligence – acutely perceiving patients’ emotional shifts, not eloquence but attentive listening.

Second, empathy and comfort are pharmaceutical humanistic care’s core instruments. Dr. Trudeau’s famous maxim – “to cure sometimes, to relieve often, to comfort always” – perfectly captures humanistic pharmacy’s essence: medications can cure some diseases, but psychological suffering requires empathy and comfort.

Third, listening is the first step in understanding patient needs. Behind patients’ medication problems often lie unspoken suffering: financial pressure, cancer’s impact on intimate relationships, and social fear after ostomy surgery. Only patients can uncover these hidden needs.

Fourth, medicine’s essence is responding to suffering, not merely curing disease. Clinical pharmacists must shift from focusing on drugs to focusing on people taking drugs, making pharmaceutical services both professionally deep and humanistically warm.

The current challenges facing hospital pharmacy reflect tensions between technology orientation and patient-centered needs. Moving toward compassionate humanistic care does not negate technology’s importance but integrates humanistic core within technical foundations: enabling clinical pharmacists to understand both drugs and people.

